# Cellular mechanical memory: a potential tool for mesenchymal stem cell-based therapy

**DOI:** 10.1186/s13287-025-04249-x

**Published:** 2025-03-31

**Authors:** Sanjay Kumar Kureel, Rosario Maroto, Kristen Davis, Michael Sheetz

**Affiliations:** Department of Biochemistry and Molecular Biology, University of Texas Medical Branch, Galveston, TX 77555 USA

**Keywords:** Mesenchymal stem cells (MSCs), Cell-based therapy, Senescence, Fibrosis, Mechanical stimuli, Mechanical memory

## Abstract

Recent studies have shown that mechanical properties such as extracellular matrix stiffness, fluid flow, weight loading, compression, and stretching can affect cellular functions. Some examples of cell responses to mechanical properties could be the migration of cancer cells from rigid to soft surfaces or the differentiation of fibroblasts into myofibroblasts. Cellular responses to mechanical changes can modify the insertion of proteins in the extracellular matrix (ECM), causing an increase in tissue stiffness with functional consequences. In general, mechanical and physical factors can affect any kind of cell phenotype in culture conditions and in vivo tissues. Cells sense mechanical stimuli by applying force and restructuring their shape and functions in response to the resistance of the stimuli. Furthermore, mechanical triggers can develop a “memory” for altering cellular plasticity and adaptation. This phenomenon is called cellular mechanical memory (CMM), a singular feature of mesenchymal stem cells (MSCs). Controlled targeting of CMM may resolve the scarcity of viable cells needed for cell based therapy (CBT) and implement studies concerning cancer research, fibrosis, and senescence. This review focusses on cells from the mesodermal lineage, such as MSCs, fibroblasts and chondrocytes, and the role of CMM as a potential target for CBT.

## Introduction

Cell-based therapy has significant potential applications in tissue regeneration and scaffolding. For example, mesenchymal stem cells (MSCs) are known for their ability to proliferate and differentiate into a variety of cells and tissue types (bone, fat, cartilage, ligaments, tendon, stroma, muscle, and neurons) [1, 2] with a wide range of regenerative capacities, including proliferative, multilineage differentiation, migratory and immunomodulatory [3].

**Fig. 1:**
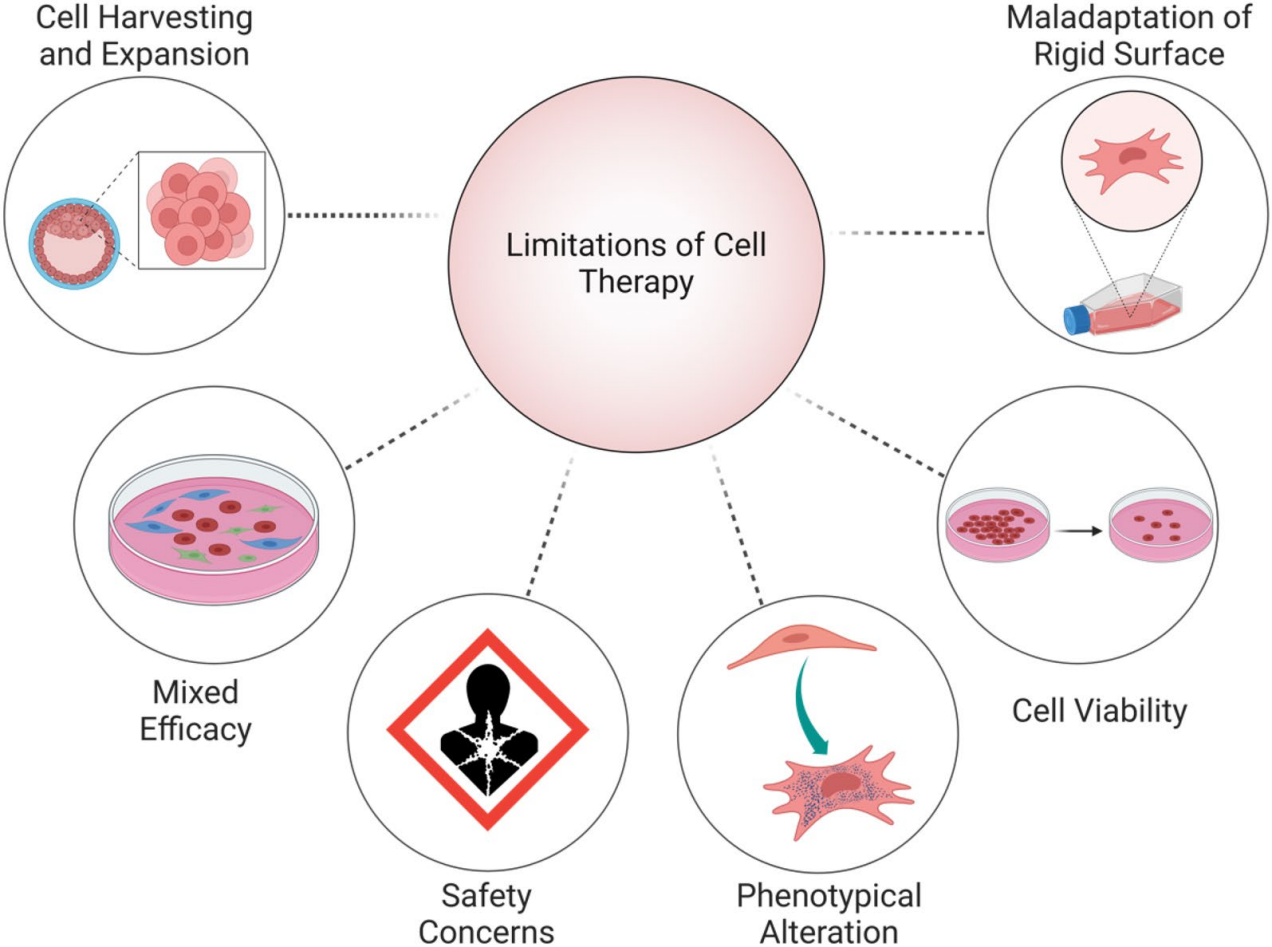
Schematic showing limitations and challenges in the implementation of cell-based therapy

To generate enough cells for different applications, cells are traditionally sub-cultured multiple times in the laboratory by trypsinization and mechanical detachment, using standard tissue culture plastic (TCP) equipment. Several studies have reported that these procedures lead to cell maladaptation to a very stiff surface that subsequently will incur in cellular dysfunctional properties [4], from loss of cellular plasticity [5–7] to reduced proliferation or altered differentiation, i.e., lung fibroblasts into myofibroblasts [8, 9] or chondrocytes into chondro-fibroblasts [10]. All these changes may also end up generating heterogeneous populations [11, 12] (Fig. 1). Therefore, developing a cell culture method incorporating time-dependent stiffening and softening properties seems imperative [13].

**Fig. 2:**
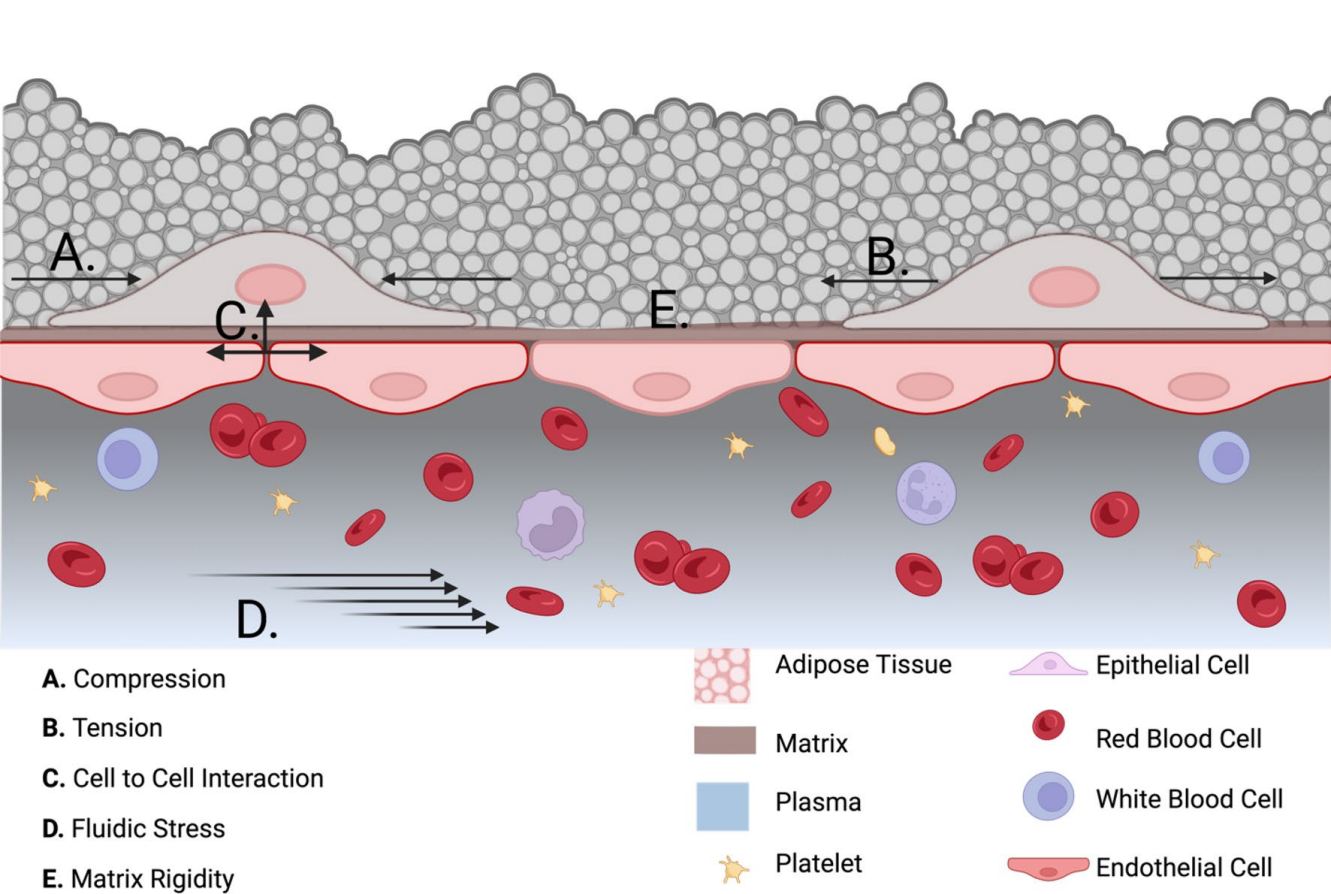
Schematic of various biophysical triggers present at the physiological tissue. Cells experience compression, tension, cell-to-cell interaction, fluidic stress, and matrix compliance. **A**. Compression on epithelial cells. **B**. Stretching or tension on epithelial cells. **C**. Cell-to-cell interaction by epithelial cells. **D**. Implies intravascular fluidic stress. **E**. Matrix compliance sense by epithelial cells

Some studies have demonstrated that mechanical forces may determine MSC lineage commitment [14–16] as well as the efficacy of tissue engraftment [17] (Fig. 2). Additionally, scientific evidence indicates that cells can “remember” past exposure to a determined trigger, i.e., cells remodel shape and modify functions in response to mechanical stimuli and maintain the remodeled status even after cessation of the stimuli. Balestrini and coworkers [8] demonstrated that lung fibroblasts cultured continuously on a physiologically stiff surface expressed fibrosis markers even when shifted to a physiologically softer surface. In contrast, lung fibroblasts cultured on a soft substrate exhibited a reduction of their pro-fibrotic potential. They coined the term “mechanical memory” to describe this phenomenon [8]. Regulation of mechanical memory, achieved by activation or deactivation, could be a strategy to generate enough viable cells for cell therapy and help to implement our understanding of the onset or progression of a particular disease (i.e., fibrosis, myocardial infarction, and cancer) [18, 19]. Studies demonstrating the existence of mechanical memory in MSCs are summarized in Table 1.

**Table 1:** Summary table of key findings associated with mechanical memory

Mechanical effector	Method used	Cell type	Disease	Implications
Intermittent uniaxial stretch [19]	Cytoskeleton/nuclear remodeling, transcriptional memory, chromatin condensation, and metabolic activity. Transmission of mechanical memory through direct cell–cell interaction	MSCs	Smooth muscle differentiation	Improved efficiency of cell therapy and tissue repair in regenerative medicine
Matrix stiffness matrix stiffness [99]	Cell area measurement ASMA staining, cell area	IPSC-Derived Cardiomyocytes (iPSC-CMs) Cardiac Fibroblasts (CFs)	Cardiomyopathy Fibrosis	Potential strategies for attenuating the impact of load-induced pathology and maintenance of mechanical memory in CFs
Encapsulation in 3D soft hydrogel [49]	3D encapsulation in HA hydrogel, PD0, PD8, and PD16 cells were encapsulated in 3D gel	Chondrocytes	Cartilage repair, 2D cell expansion	Regeneration procedure and design of biomaterials
Substrate stiffness [4]	Five days on stiff then moved to soft	OSCC cell lines, Cal27	Oral Lesion, cancer migration and invasion	Tumor progression
Botulinum hemaglutinin (HA), blocking agent of E-cadherin [20]	Single dose of HA and repeated dose of HA, HA based synchronization mediated mechanical memory. HA treated cells differentiate into hepatocytes but suppresses others. HA treated cells show cytoplasmic YAP	iPSCs	NA concentration and exposure time retain MM and hence pluripotency of hiPSCs	Cell therapy, drug discovery, stemness maintenance of ihPSCs
Substrate stiffness [9]	PDMS (2–180 kPa), 35 kPa PDMS maintains tendon. phenotype without activation of fibrosis	Human tendon derived stroma cells	Tissue homeostasis	Large scale cell expansion
Substrate stiffness and duration of treatment [50]	GelMA hydrogel, P0-P3 on soft then switched to stiff, 12–145 kPa	hPDLSCs	Regeneration of periodontal tissue, Integrin linked kinase is involved in osteogenic lineage	Periodontal tissue engineering
Substrate stiffness [34]	PEG and NIPAm, Cells primed on varying stiffness for 7 days, 100 Pa- 1000 Pa	Hepatic satellite cells	Fibrosis, trypsinization causes increase in alpha SMA, and fibrosis	Cell based therapy and wound healing
Substrate stiffness [11]	ASCs lose adipose potential on TCP, retained adipose potential when primed on stiff then moved on soft, 100 kPa	Adipose tissue derived Stem cells (ASCs)	Fat grafting and soft tissue generation, nuclear area and LINC protein nesprin2 are the key player	Stemness maintenance of SCs for tissue therapy
Stiffness [8]	Soft to stiff prevents activation of myofibroblasts, 5–10 kPa and 16 kPa	Lung airway fibroblast	Lung fibrosis	Fibrosis
Stiffness 0.5 kPa and 40 kPa [44]	MSCs on stiff for 10 days then move to soft shows osteogenic (RUNX2 and YAP in nucleus), Soft-stiff and stiff-soft at varying duration	Human MSCs	Stemness maintenance	Regulation of MSCs Differentiation
Cyclic loading [101]	Stretch and loading induced condensation of chromatin and calcium signaling, Duration of loading and degree of strain causes MM	Human MSCs	Stemness maintenance	–
Geometric cues [23]	Star and oval shape, Soft-stiff Stiff-soft	MSCs	–	MSCs fate regulation
Soft and stiff sub-strate [26]	miRNA-21, 5 and 5 and 100 kPa, Stiff surface activates miRNA21 and priming on soft then KO of miRNA prevents MSCs fibrogenesis	MSCs	Fibrotic program	Prevention of MSCs to form fibrosis
Substrate rigidity [4]	50 kPa and soft, Priming, Rewiring	MCF10	Cells primed on stiff showed higher migration than that of soft	Potential migration and cytoskeletal changes
Computation model, substrate stiffness [43]	Priming	MSCs	MSCs primed on soft surface retained multiple lineage commitment	–
Substrate stiffness [7]	Soft and stiff surfaces, Soft surface priming cells delays fibrosis	ASCs	Fibrosis	Fibrosis and elbow injury
Dynamic hydrogel system [37]	Varying surface exposure, Ca + 2 signaling modulate rewiring	MSCs	Differentiation potential	Differentiation

In this review, we examine recent findings regarding the molecular basis of CMM and current factors involved in CMM testing and regulation, focusing on three models: MSCs, lung fibroblasts, and cancer cells. We also discuss how CMM can generate enough cells for cell-based therapy. Finally, we analyze the limitations, challenges, open questions, and perspectives of using CMM to enhance cell based therapy (CBT) success in mitigating susceptible diseases.

## Biological basis of cellular mechanical memory

The novel concept of mechanical memory may substantially impact the scientific understanding of how changes in the physical environment of the cell affect its long-term outcomes. The term memory is difficult to define and measure in biological research. However, we can apply some criteria that allow for a measurable determination of whether a cell or an organism can remember based on retrieval and accurate application of information from a past event. Firstly, memory is time-dependent, meaning it is to be recalled from a previous point in time. Thus, it refers to the present information of an experience or event that occurred in the past. Secondly, to be classified as memory, the knowledge of the experience must be retrievable. If an event occurred, but it cannot be retrieved by the cell/organism, then it cannot be remembered. Lastly, memory must have a certain degree of accuracy. Accuracy within memory can be expressed as the correlation of the previous experience to the action associated with the recall.

**Fig. 3:**
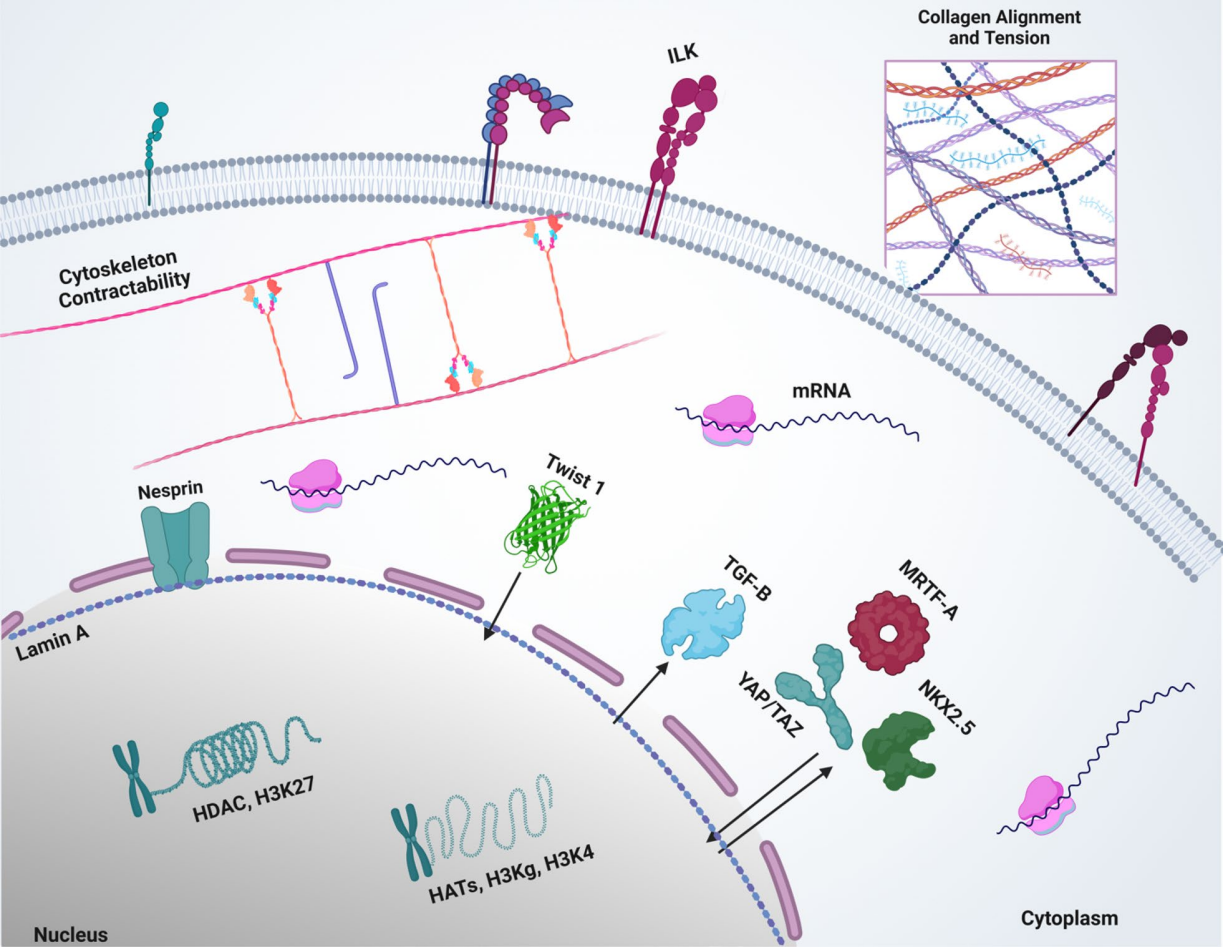
Schematic of intracellular reactions triggered by Mechanical Memory. When a cell experiences a mechanical stimulus, Twist 1 enters the nucleus, TGF-B exits the nucleus towards the cytoplasm, and MRTF-A. NKX2.5 and YAP/TAZ enter and exit the nucleus

It has been well demonstrated that external forces can remodel cell structure and functions and attain a new temporal status in cells and tissues. Total or partial regain of the original status depends on the exposure time, types of stimuli, and frequency of exposure [31]. For instance, in reversible CMM cells, they recover their original state, whereas in irreversible CMM cells, they experience partial recovery. After experiencing the first stimuli, cells may retrieve information to adapt and survive another occurrence. Nevertheless, many questions remain unknown, such as how cells store and recall the experience when they reencounter the same stimulus and how cells use CMM during a different stimulus. In this section we examine the role of various cellular and molecular structures and signaling pathways in acquiring CMM (Fig. 3).

### Role of extracellular matrix and cytoskeletal organization

A 3D microenvironment promotes crosstalk between cells and the ECM, which consists of collagen-I and laminin. Almeida and coworkers [18] demonstrated that cells can transfer memory to surrounding cells and the following generation by activating tension and aligning collagen filaments. Collagen-I modifications can activate Yes-associated proteins (YAP), a hallmark of CMM acquisition by the ECM. Cells communicate with surrounding cells via cell-cell junction proteins, i.e., E-cadherin. Kim and coworkers [20] used cadherin-E blockers to alter cytoskeleton rearrangement via activation of YAP and phosphorylation of myosin light chain (MLC). However, how E-cadherin inhibition regulates the acquisition of CMM remains elusive.

Mechanical forces are tested and transduced through adhesion proteins, mainly integrins, focal adhesion proteins, and kinases. The mechanotransduction process involves altering integrin conformation and kinase linkage. It has been demonstrated that integrin linkage kinase (ILKs) can activate YAP and establish CMM [21]. In this case, the pathway described for CMM acquisition was the Akt signaling pathway and recall, possibly via phosphorylation of focal adhesion kinases (FAKs) through Rho/ROCK and cytoskeleton rearrangement [19]. Still, how the adhesion complexes are involved in CMM storage and recall needs further investigation.

Cytoskeleton plays a crucial role in force transmission and transduction. Actin, intermediate filaments, and microtubules are the main components of the cytoskeleton structure. Actin is a tensile-bearing element, whereas microtubule is a compression element. The intermediate filaments have both tensile and compression abilities. Cells sense external forces by applying force through the actin-myosin contractile unit. Cellular contractility determines cellular shape and size via Rho/ROCK downstream molecules. Therefore, actin cytoskeleton and contractile units contribute to the CMM storage and recall processes [22, 23]. It looks like mechanical memory is stored by activation of YAP through a combination of actin-myosin contraction units and cytoskeleton rearrangement [4].

Mechanical stimuli are transmitted to the nucleus through several cytoskeleton elements, a net of proteins formed by Nesprin and nuclear-cytoskeleton linker complexes (LINC) that connect to the nuclear protein Lamina. The role of LINC complexes in determining the nucleus shape has been studied by Berger and colleagues [11], who uncovered that mechanical memory could be established by altering the nuclear size and shape based on conformational changes of Nesprin. The nuclear envelope tension can activate nuclear pore channels, furthering the impact on the chromatin state, specifically de-condensation, protein diffusivity, and development of mechanical memory after mechanical stimuli [24]. Although several studies have indicated that the cytoskeleton and contractile units play a vital role in CMM storage and recall, some questions still need future investigation. For instance, how transcription factors such as YAP/TAZ are regulated by the cytoskeleton assembly? How dynamics and turnover of actin and microtubules contributes in the CMM storage and recalling processes?

### Epigenetic modifications

Many studies have repeatedly shown that mechanical memory can be acquired under the persistent influence of epigenetic modifications via mechanical signals [12, 25, 26]. Stem cells sense physical stimuli and consequently remodel their shape and functions. They can also remember past stimuli, even when exposed to a new environment. In this section, we discuss the role of epigenetics in maladaptation and mechanical memory establishment in a stiff environment (Fig. 3).

Mechanical cues can regulate chromatin accessibility and remodeling [27–29]. A recent report has indicated that long-term MSC culture on stiff surfaces could alter MSC functions via epigenetic modifications [12]. Several studies have shown that epigenetic changes may encode a long-term mechanical memory in epithelial cells. For instance, histone modifications, including methylation and acetylation of histone-by-histone acetyltransferases (HATs), histone deacetyl transferases (HDACs), DNA modifications, and non-coding RNAs. Other studies have addressed the role of epigenetics in regulating stem cells’ fate [30–33]. For example, cyclic stretching can lead to chromatin condensation through YAP activation. Calcium signaling and adenosine triphosphate (ATP) release can also activate YAP and CMM [25]. Fan and colleagues [34] showed that HATs and HDACs could influence chromatin de-condensation. Histone proteins H3K9, H3K4, DHACs, and H3k27 have also been involved in condensation and de-condensation of chromatin, contributing to CMM activation. Thus, protein lifetime and 3D chromatin organization have been reported to activate CMM upon applying a sustained mechanical stimulus [35]. Hussien and colleagues [9] tested the role of chromatin organization and distribution of CMM. They discovered that surface stiffness was activated by SUZ1/2 and EZH2, which formed the PRC2 complex and altered chromatin organization in heterochromatin. Upon alteration in chromatin structure, mechanical stimuli changed the size and distribution of the histone trimethylation protein H3K9me3. For instance, late cell passages exhibited H3K9me3 in puncta form, whereas early cell passages expressed H3K9me3 mostly near the nuclear lamina. These reports highlight the role of histone proteins in the storage and retrieval of CMM.

Other reports have indicated that chromatin condensation and de-condensation could influence the functionality of stem cells. Examples are stretch-mediated chromatin condensation, described to preserve MSCs’ differentiation in smooth muscle [36], or de-condensation, which favors nuclear alteration and DNA damage responses [24].

Besides chromatin modifications, non-coding RNAs, such as microRNA-21 (miR-21), have been demonstrated to participate in CMM acquisition and long-term storage. MicroRNAs (miRNAs) are part of the components of the epigenetic modification machinery. Investigating the role of miRNAs in CMM activation, Wei and colleagues [37] uncovered that miRNA-21 was overexpressed in response to mechanical stimuli. Another vital contribution to the field was the discovery by Li and coworkers [26]of the focal adhesion activation mediated by ROCK protein, myocardin-related transcription factor A (MRTF-A). MRTF-A entered the nucleus and was bound to the serum response factor (SRF), forming a complex brought to the proximity of CarGBOX [26], which then activated miRNA-21 via Smad7. This finding suggested that miRNA-21 is an essential regulator of CMM.

Furthermore, CMM was erased by knocking down miR-21 in the same preparation. It seems that miR-21 plays a critical role in maintaining long-term mechanical memory, and its regulation could be used to prevent fibrotic differentiation of MSCs [26]. In contrast, other studies have shown an inhibitory effect of miR-21 in MSCs related to inflammation and senescence associate secretion pattern (SASP) [38]. Thus, epigenetic regulation of CMM might be used as a novel approach to generate MSCs for CBT.

### Role of the Hippo signaling pathway

YAP/TAZ, TEAD1-4, MST1/2, and LATS1/2 are critical components of the Hippo signaling pathway (HSP), a key regulator for disease development, regeneration, and aging or disease progression [39]. It is well documented that dysregulation of the HSP leads to the development of cancer, immune, and age-related diseases [39, 40]. From a functional point of view, HSP has been referred to as an alternate between two states, “on” and “off.” When the HSP is turned off, YAP/TAZ is activated and translocated into the nucleus, regulating gene expression via binding to TEAD1. However, YAP/TAZ becomes phosphorylated and degraded when the HSP is turned on. Upstream regulators, such as cell density, mechanical or  geometric cues, stress, and polarity, can determine the HSP “on and off” status and affect the transcriptional activity and control over cell proliferation, differentiation, and self-renewal [40].

Many studies have shown that YAP/TAZ activity and mechanical cues are tightly related and can influence proliferation, differentiation, and survival via HSP. For instance, low ECM stiffness can activate upstream molecules of YAP/TAZ and LATS1/2 [18, 37, 41]. Other studies have shown that cells get inflated and spread when YAP is in the nucleus [42]. Interestingly, the translation of YAP to the nucleus is retained even after exposing the same cells to soft surfaces, giving the concept of developing mechanical memory. This phenomenon has also been noticed in cancer cells. YAP activation in the nucleus increases invasiveness and migratory potentials in cancer cells. Thus, YAP activation and nuclear translocation can play a crucial role in stem cells.

Price and coworkers [35] have computationally shown that actin polymerization can promote YAP migration into the nucleus and influence F-actin expression via Rho- pathways. *In vitro* cell studies have shown that translocating YAP and MRTF into the nucleus due to mechanical stress can activate RUNX2 and genes associated with cell survival. Fibroblast differentiation to myofibroblast is marked by the overexpression of alpha-smooth muscle actin (αSMA). Some studies have shown that YAP-MRTF and serum response factor (SRF) regulate αSMA expression. The Smad pathway, mediated by the SRF and TGF-β, regulates αSMA [12, 37, 43].

Persistent exposure to mechanical stimulation can also lead to YAP/TAZ activation, followed by aSMA activation and ending up in mechanical memory activation [4, 8].. Prolonged loading can activate YAP/TAZ sustained nuclear localization for days, whereas transient mechanical loading the translocation will last only several minutes. Transient and sustained nuclear localization can contribute to short-term and long-term CMM. In response to transient mechanical stimulation, MRTF-A is released by G-actin, promoting its nuclear translocation. Nuclear MRTF-A can bind to SRF and form a complex that regulates cytoskeleton and adhesion complexes. This positive reinforcement of the cytoskeleton and nuclear structure will help to establish mechanical memory. Therefore, positive feedback can reinforce the cytoskeleton and nuclear structures and regulate the activation and persistence of mechanical memory even after the stimuli have been removed [44, 45]. In addition to YAP/TAZ, Smooth muscle receptor NKX2.5, Twist1, and transforming growth factor-beta (TGF-beta) can also shuttle between the nucleus and cytoplasm in response to mechanical stimulus and contribute to mechanical memory development [26, 37, 46]. Thus, relocating transcription factors, coactivators, and signaling molecules to the nucleus and cytoplasm is the critical hallmark of mechanical memory acquisition.

In summary, prolonged changes in actin cytoskeleton contractility, translocation of transcription factors, chromatin condensation and de-condensation, miRNA, and many associated signaling pathways by mechanical stimuli offer a novel approach as mechano-reprogramming of stem cells and fibroblasts and thus provide an opportunity to develop a method of stem cell expansion for CBT.

## Activation, regulation, and assays for cellular mechanical memory

###  Activation of CMM

**Fig. 4:**
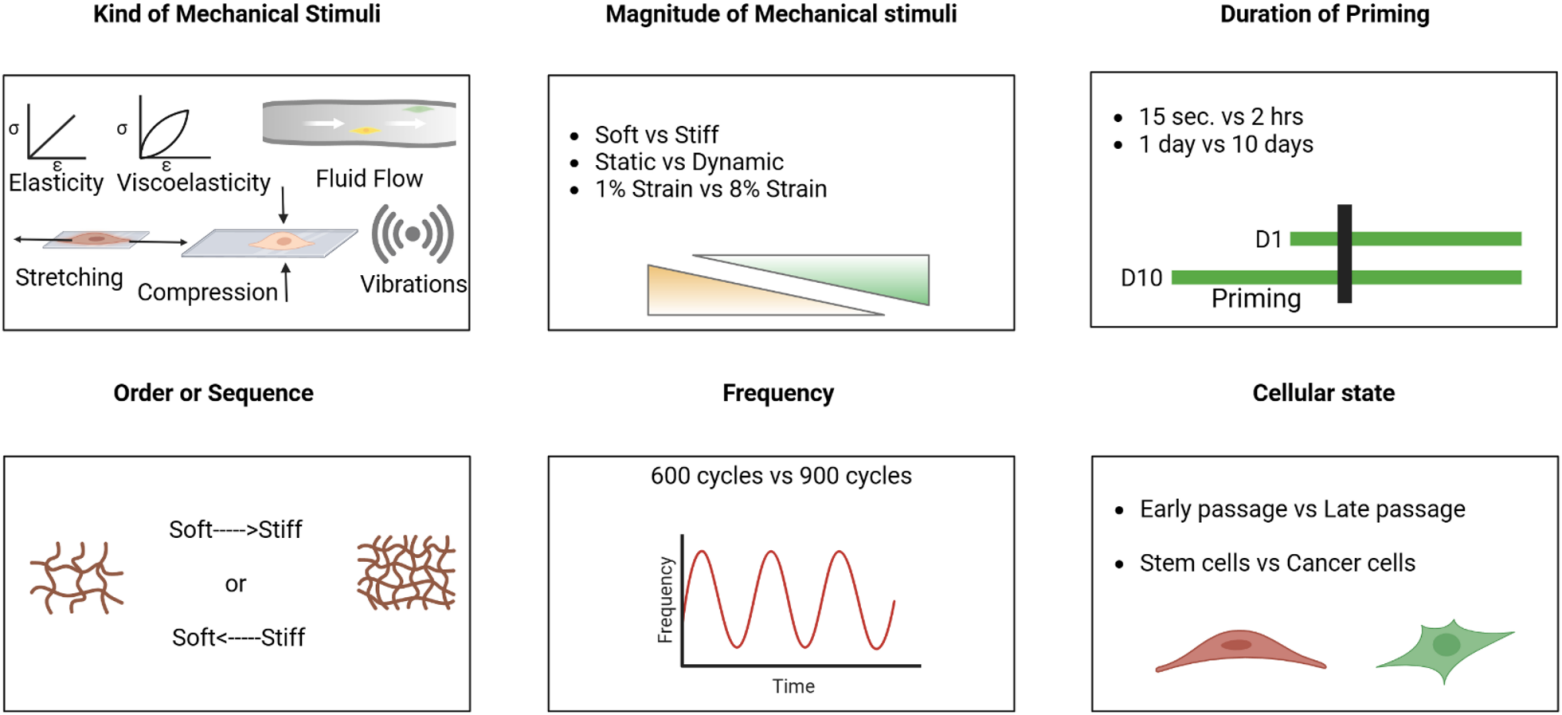
Schematics of various modulators of cellular mechanical memory. These modulators impact the intensity and duration of CMM.

CMM activation is the process by which mechanical information is transferred to the cell. The first step in CMM activation consists of cell priming, which is culturing cells on two surfaces of different rigidity for a different duration of time (Fig. 4). Nevertheless, cell priming can also be achieved using a dynamic substrate, i.e., elastic modulus where the substrate rigidity can be changed. Several studies have been referred to in the literature for using these strategies. For example, MSCs were primed on 03 kPa and 40 kPa PA gels, activating CMM within a stiff environment [46]. Human tendon stroma cells were isolated and cultured on 2, 5, and 180 kPa polydimethylsiloxane (PDMS) coated surfaces until they reached 80% confluency [46] CMM was instilled in MSCs by repeated 3% dynamic stretching (loading) for different durations [37]; CMM was maintained between passages of MCF10A cells which were primed on heat-responsive and stiffness tunable hydrogel from day 1 to day 7 [47]. Local stress applied to integrins for 2 minutes and 10 minutes resulted in the activation of nuclear pore complexes and the development of CMM [36]. Alginate-based dynamic hydrogel instilled CMM in MSCs [42] and MSCs cultured on nanohybrid scaffolds with stiffness relaxation properties [47].

Cell rewiring in an opposite mechanical environment, followed by priming, is another approach to induce CMM activation. Rewiring involves swapping or switching the mechanical environment of cells after priming. For instance, lung fibroblasts were primed on 5 and 100 kPa PDMS for 3 weeks and then switched to opposite stiffness for 2 weeks [47]. Priming on a physiological soft surface protects lung cells from fibrosis. In a seminal study, Yang and coworkers primed the MSCs on TCP for 1, 5 and 10 days. After that, the cells were switched to a soft polyethylene glycol (PEG) hydrogel for 7 days. They also tested a phototunable hydrogel for *in situ* substrate softening and stiffening for 1, 3, 5, and 10 days [44]. MCF10A and mammary epithelial cells (MECs) were primed on methacrylate hyaluronic acid hydrogel, a dynamic hydrogel, for 10 days then the hydrogel was switched to stiff for another 10 days [48]. MCF10A cells were primed on 0.5 and 50 kPa of PA-gels for 3 days and then switched to the opposite stiffness for 12 hours [48]. Squamous carcinoma cells (SCs) were primed on 0.5 and 20 kPa PA-gels for 5 days and then rewired on opposite stiffness for one day [25]. Thus, priming and rewiring on constant stiffness and dynamic substrates have been used in CMM activation. Mechanical cues, pharmacological drugs, and geometrical cues [23] have also been used to activate cellular mechanical memory (Table 2). Pharmacological drugs such as *botulinum hemagglutinin* (HA), an E-Cadherin blocker, have also been used to synchronize hiPSCs, which helped to preserve differentiation capacity into hepatocytes by activation of CMM [20]

**Table 2:** List of pharmacological modulators of CMM

MM modulators	Functions
Leptomycin B [44]	Nuclear export inhibitor
miRNA-21 mimic [37]	Erases mechanical memory
Botulinum hemagglutinin (HA) [20]	Cadherin blocker, induce homogeneity in populations
JIB-4. Pitstop. Wheat germ agglutinin. Chaetocin. TSA and Chaetocin [24]	Histone demethylase inhibitor, JIB-4 condenses chromatin. Nuclear pore complex permeability barrier disrupter. Nuclear pore blocker. Methyltransferase inhibitor which decondenses chromatin. Both decondense chromatin
ML324, KDM4 inhibitor. Y27632, contractile inhibitor. GSK343, Enzyme EHZ2 inhibitor TSA, Histone deacetylase blocker. Apyrase, ATP blocker. Flufenamic acid and Oligomycin. BAPTA. GsMTx4, piezo 1 blocker [25, 49]	Enzyme EHZ2 inhibitor TSA, Histone deacetylase blocker. Apyrase, ATP blocker. Flufenamic acid and Oligomycin.BAPTA. GsMTx4, piezo1 blocker. KDM4 Demethylase inhibitor. Inhibits chromatin condensation. Prevents chromatin condensation. DL does not cause changes in CCP in the presence of Apyrase. ATP inhibitors. Calcium Chelator. Prevents condensation of chromatin
Blebbistatin, myosin blocker. LPA. Perifosine, AKT blocker. FAK inhibitor-14 [19]	Blocks migration potentials. Increases migration potential. Blocks single cell and collective cell migration. Enhances detachment and inhibits cell adhesion
Verteporfin, YAP inhibitor. Galunisertib, TGF-beta inhibitor [48]	Inhibits growth, autophagy, and induces apoptosis. Inhibits the growth of tumor cells
Glutathione and 1.7 mM. LAP [12]	In situ softening hydrogel
CCG-1423, MRTF-A blocker [26]	MRTF-A is found in cytoplasm, Enters nucleus during stress
jasplakinolide [103]	Actin polymerization activator
U0126, MEK inhibitor [104]	MEK1/2 kinase inhibitor

###  Regulation of CMM

A critical aspect of CMM research is identifying specific factors that can affect the intensity and duration of CMM (Fig. 4). One of these factors is the duration of priming time***.*** It has been shown that exposing human mesenchymal stem cells (hMSCs) to a soft substrate for a short period can instill a reversible CMM, whereas long duration can cause irreversible CMM [12, 44]. Similarly, the exposure time to mechanical cues, such as local stress [24] and dynamic stiffening, [48] can affect CMM and outcomes. The high stiffness of the priming substrate can promote dysregulation of MSCs fate [49], fibrosis activation [4, 26], and migration and invasion of cancer cells to softer regions [18, 19]. Priming on a stiff substrate followed by switching to a soft substrate reduces adipogenic differentiation by 50% in adipose-derived stem cells [23]. Priming on soft substrate has also been shown to prevent lung fibroblasts [18] and adipose-derived stem cells (ASCs) [11] from developing a fibrotic phenotype. Also, MSCs soft primed have been related to improving transplant efficiency [26]. Another factor to consider is the frequency of exposure of the cells in the culture to a determined stimulus. In this regard, repeated exposure or higher frequency of exposure can regulate CMM by favoring the alteration of chromatin structure [25].

Differences in the status of the cells used in CMM studies can also affect CMM activation and regulation. For instance, cardiomyocytes derived from induced pluripotent stem cells (iPSCs) developed CMM in 24 hours via microtubule-dependent compression. However, it took 7 days for cardiac fibroblasts to develop CMM via actin-dependent pathways [13]**.** Another example is the chondrogenic differentiation and doubling population of chondrocytes, which have been shown to benefit from priming on TCP and encapsulation in 3D hydrogel [49]**.** Therefore, cell status can influence mechanical memory and cellular outcomes.

Aside from mechanical stimuli, regulation of miRNA-21, NKX2.5, YAP/TAZ, and epigenetic modifiers such as HDACs and HATs [12], histone proteins H3K9me3v, regulators of cytoskeletal contractility, and chromatin modifiers have also been used to regulate CMM [44], [46]. Activation and recovery of CMM requires Akt and FAK signaling pathways; thus, regulating these pathways should help to manipulate CMM [19]. Nonetheless, there are other mechanical stimuli or combinations to explore, such as fluid flow, shear stress, pressure gradient, vibration, spatial constriction, crowding, ECM depositions and degradation, optogenetics, or sound waves.

### Cellular mechanical memory assays

**Fig. 5:**
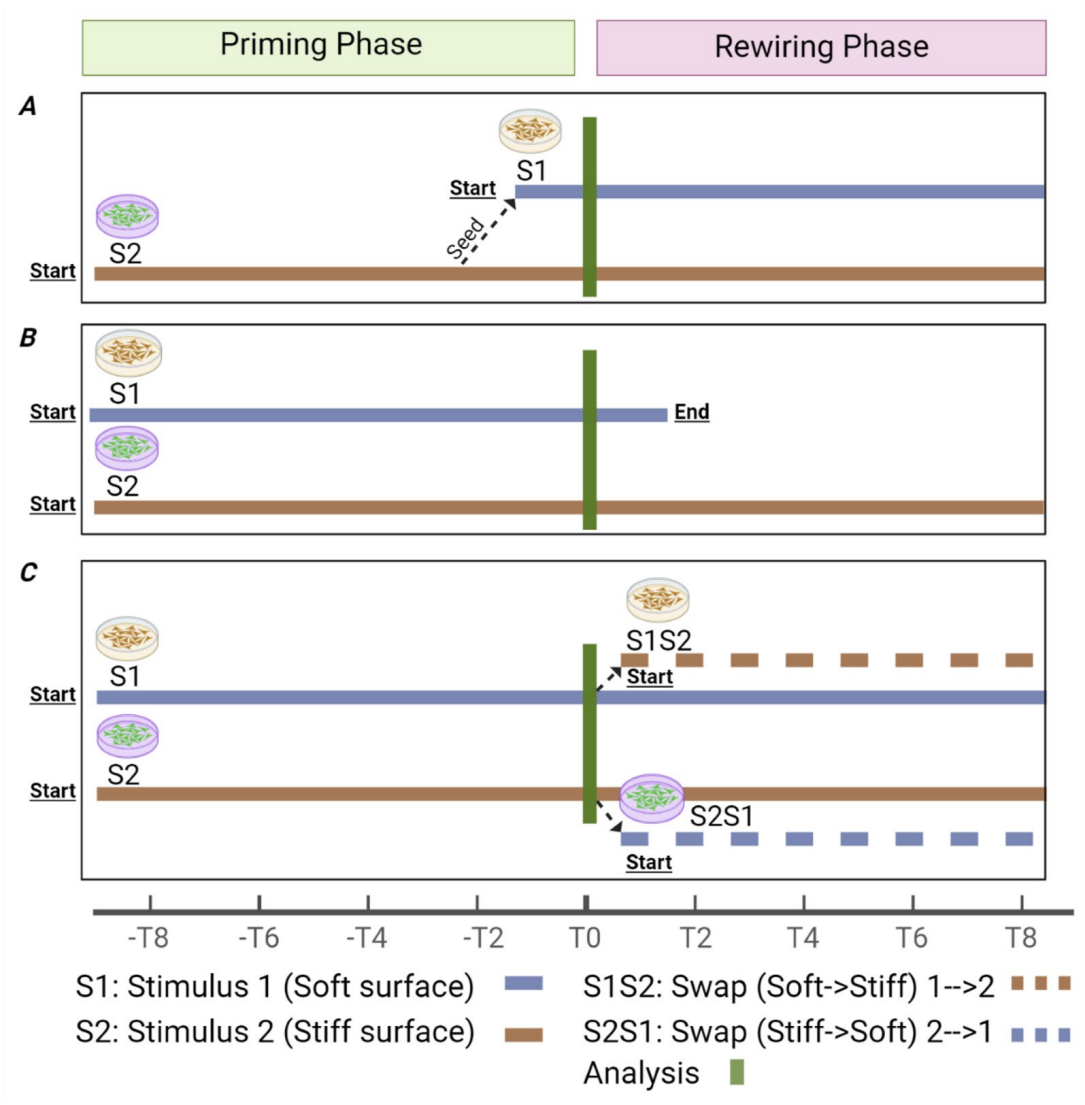
Schematic demonstrating the mechanistic of priming and testing phase of cells, analysis point, and swapping cells to soft and stiff surface. **A**. S2 cells can be cultured and grown on stiff substrate for five days, then analyzed or continued growing for the remainder time of the experiment. S2 cells can be seeded on S1 and begin growth on day 4, analyzed on day 5. Cells can continue to grow for the remainder time of the experiment. **B**. S1 cells can be cultured on soft whereas S2 cells are cultured on stiff surface. Both cells’ samples can be analyzed on day 5. S2 cells can continue to grow for the total length of the experiment. **C**. S1 cells are cultured on a soft substrate whereas S2 cells are cultured on a stiff surface. In both cases cells can be analyzed on day 5. Then S1 can be seeded on stiff (S1S2), and S2 can be seeded on soft (S2S1). The four types of cell samples can continue growing for the remainder length of the experiment to test if the cells remember their past environments and adapt to the new environments

A significant challenge in CMM research has been and still is how to measure it. There is no direct measurement tool for CMM. However, various approaches have been used to study CMM (Table 3). To date, translocation, and expression of signaling molecules and transcription factors have been used to follow up on the activation and deactivation of CMM (Fig. 5). For instance, phosphorylation of YAP induces its translocation to the nucleus, which is considered a CMM activation marker [4, 11, 20, 25, 26, 44, 50]. Release of NKX2.5 [46] from the nucleus, overexpression of microRNA-21 [26, 37], variations in nuclear shape and size [26, 37], changes in chromatin structure [10, 25], DNA methylation [34], expression of H3K9me3 [38, 49], expression of fibrosis markers, and phenotypical or differentiation changes [11, 50] are all currently used as indirect markers of CMM. However, there is an urgent need for the development of a universal CMM assay.

**Table 3:** List of assays used to study cellular mechanical memory

Experiment design	Cell type	Assays	Pathways
Cytoskeleton/nuclear remodeling, transcriptional memory, chromatin condensation, and metabolic activity. Transmission of mechanical memory through direct cell–cell interaction [36]	MSCs	Cell alignment, F-actin distribution, nuclear shape, chromatin condensation, gene expression, mitochondrial morphology, ATP production, proliferation, and differentiation	Activated by amplitude frequency and duration of stretch. Regulated by cytoskeleton, nucleus, chromatin, and metabolism
Cell area measurement [99] ASMA staining, cell area	IPSC-Derived Cardiomyocytes (iPSC-CMs) Cardiac Fibroblasts (CFs)	Microtubule-dependent induction and maintenance of mechanical memory in iPSC-CMs Actin dependent induction and maintenance of mechanical memory in CFs	Activated by a-smooth muscle actin, regulated by fibrosis
UV induced photodegradable hydrogel; cells were exposed to in situ variable stiffness different culture time [44]	hMSCs	Differentiation markers and YAP localization in cytoplasm and nucleus	YAP, RUNX2, pparγ, OCN
In situ softening and stiffening using phototunable alginate hydrogel [37]	MSCs	microRNA-21 and osteogenic differentiation markers	microRNA, OPN, and RUNX2
Effect of priming, cells were cultured on nanofiber scaffold [105]	MSCs	Makers of proliferation, differentiation, and ECM production	Collagen density and alignment, pparγ, and RUNX2
Computational method [106]	–	Mechanical feedback and cell division	–
Cells were passaged at p1, p8, and p16 then encapsulated in 3D HA-PEGDA hydrogel [49]	Chondrocytes	Chondrogenic genes, nuclear shape and chromatin organization	Expression of H3K9me3, nuclear area
Computational approach [35]	–	Studies The effect of positive enforcement in mechanical memory	Duration and stiffness of Priming and recovery phase impact CMM
Computational [43]	–	Simulation of gene regulatory network for predicting the mechanism of CMM	Duration of primary phase and reseeding MSCs on soft preserved all lineages
Cells were cultured on 0.5 and 50 kPa gels and PDMS for priming. Does priming on stiff surface cause increased migration [4]?	MCF10A	Migration speed and Cyto/Nuc YAP, Actin alignment	FAs, pMLC, Actin, YAP
Cells were cultured on 0.5 and 20 kPa PA gels [19]	Lung Fibroblast	Expression of contractility molecules, cell-cells and cell-ECM junction proteins	NMM2, FAK, E and N cadherin
Effect of priming, switching for different duration from soft to stiff and vice versa on activation of MM fibroblast in vitro and in vivo. 5, 100 kPa and TCP were used for the study [26]	MSCs	Short and long-term MM regulators	Collagen density, YAP/TAZ, miRNA-21, alpha SMA
PA gels with embedded collagen (MMMS); heterogeneous substrate and homogeneous ligand. 0.3 and 40 kPa gels were used. Studied the effect of surface of heterogeneous stiffness on CMM and cell fate [46]	MSCs	Inducing MM using NKX2.5. and substrate of heterogeneous stiffness but homogeneous ligand	NKX2.5, Nuclear localization signals (NLS), Alpha SMA
Substrate stiffness and cell shape on MM. 0.5–40 kPa PDMS [23]	MSCs	Early and late osteogenic and neurogenic diff markers	RUNX2, OPN, MAP2, Beta3 tubulin
Study synchronized behavior driven MM using HA, E-Cadherin blocker [20]	Hepatocytes and iPSCs	Hepatocytes specific markers and differentiation markers	Cyto/nuc YAP ratio, Actin E-cadherin, integrin expression
Bioinformatics approach. Mechano-variant PDMS substrate. 2 kPa, 35 kPa, and 180 kPa PDMS [9]	Human Tendon-derived stromal cells	PI3/AKT and ERK1/2 pathways are central to stiffness sensing. Transcriptomic comparison	Cell counting and cell area, population doubling and doubling time
Effect of various loading on MM [25]	MSCs	Nuclear deformation and chromatin condensation	YAP, Nuclear deformation index (NDI), and chromatin condensation parameters (CCP) state
Swapped the substrate [50]	hPDLSCs	Shuttling of YAP and differentiation markers	Nuc/cyto YAP ratio, RUNX2 and OCN
Compared trypsin and thermally driven detachment. PEG-NIPAm Hydrogel [34]	LX-2 Cells	DNA methylation and fibrosis markers	Actin, alpha SMA, fibronectin, H3K9me3, Gamma H2XA foci
Cells primed on 1 kPa gel shown less fibrosis and improved wound healing [7]	Adipose derived MSCs	Cytoskeletal change and fibrosis markers	α SMA, cell area nuclear size
Effect of swapping of stiffness on MM, PDMS [11]	ADMSCs	Change in Differentiation markers	Pparγ, RUNX2, YAP
Effect of swapping environment on fibrosis [8]	Lung fibroblast	Markers of fibrosis	α SMA and number of myofibroblasts
Effect of priming on soft and stiff surface on cell invasion and migration potentials. Cells were primed for 5 days on soft and stiff surfaces. 80 Pa and 16,000 Pa, PA gel was used [18]	MCF10A Breast epithelial cells	Cellular invasion and change in ECM structure due to priming	Actin and collagen alignment and distribution

How cells store and retrieve mechanical information is another area for further investigation. During the past few years, there have been significant advancements in analytical and computational approaches, including artificial intelligence (AI) [51], generative AI [51–53], computational and bioinformatics [54–57], and software developments to predict the 3D structure of proteins [58, 59]. Moreover, super-resolution microscopy [60, 61], single-cell sequencing [57], programmable hydrogels [62, 63], multi-omics [64, 65], Hi-C technology [66–68], and a combination of deep learning [69, 70] and molecular biology have facilitated some understanding of biomolecules’ intricate organization, interaction, and dynamics with their surroundings. For example, super-resolution microscopy has allowed the study of transcriptional kinetics at the protein level [71]. Single particle analysis has helped the study of transcriptional factor kinetics [71, 72]. Oligopaint technologies have improved the study of simultaneous tracking for chromatin landscape and gene sequencing [73] by combining imaging of real-time gene expression and chromatin accessibility [74, 75]. Applying these techniques along with mechanical properties measurements to cells and tissues, *in vitro* and *in vivo* contexts, still holds considerable promise in enhancing our understanding of transcriptional regulation and CMM mechanosensing.

## Cellular mechanical memory in health and disease

**Fig. 6:**
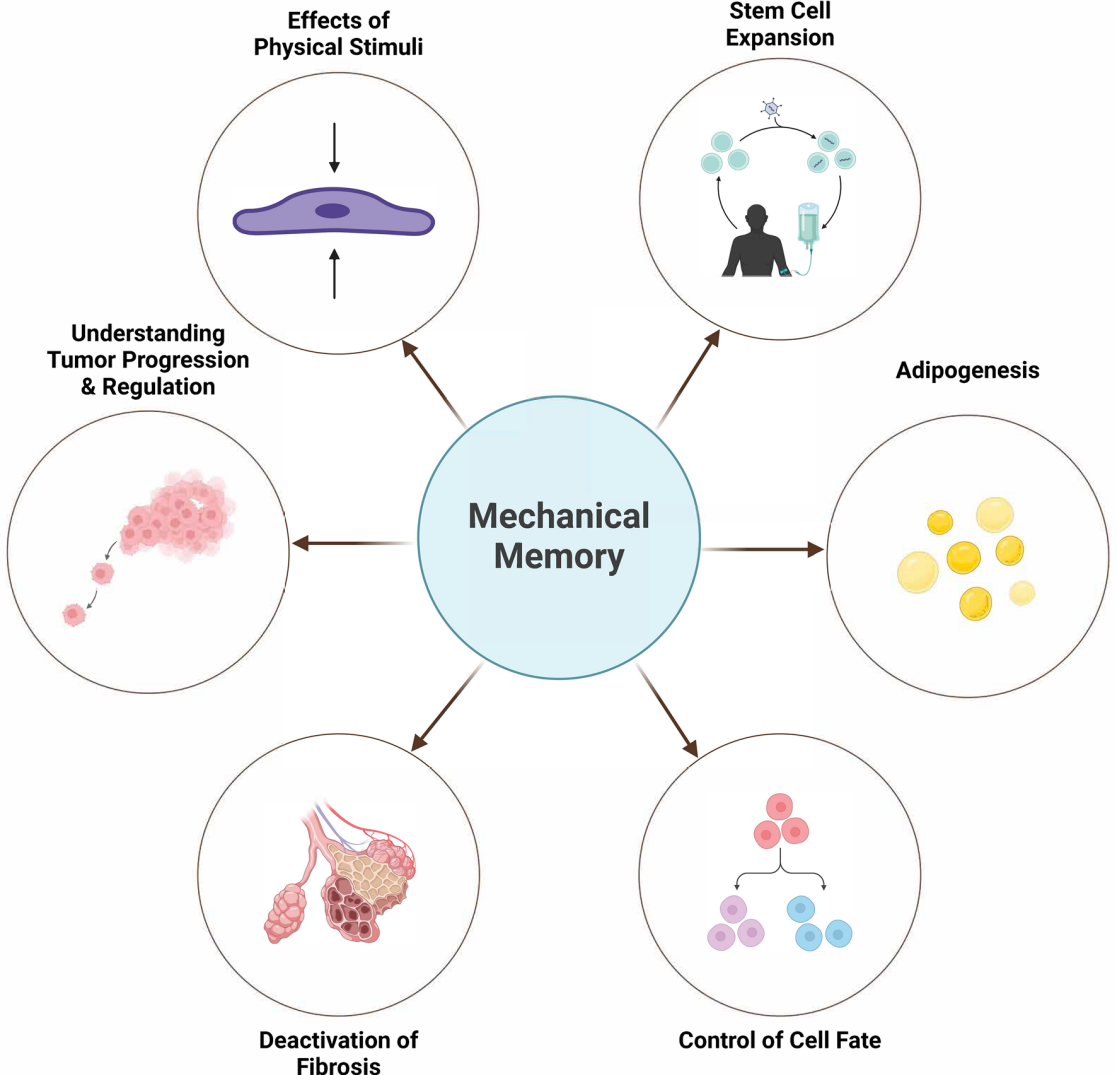
Schematic representation showing benefits of mechanical memory among current challenges and limitations within healthcare and cell therapy techniques

For health and disease conditions, cells and tissues encounter diverse mechanical challenges to maintain homeostasis, e.g., from blood clotting to tissue scaring, from tumor growth to cancer metastasis, or from tissue injury to wound healing. All these examples converge in a common question about how cells sense and store mechanical signals and how they apply such information for survival through adaptation to a new mechanical setting. In this section, we’re discussing the potential application of CMM to cell-based therapies, diseases, regeneration, and tissue engineering (Fig. 6).

### Maintenance of a native phenotype during in vitro expansion for CBT

Using a CMM approach to reset maladapted cells due to exposure to a stiff surface could improve the potential differentiation of any kind of cell, mainly stem cells. For example, expanding ASCs on a soft substrate resulted in a higher adipogenic differentiation potential compared to a stiff surface [11]. Stem cells can maladapt on stiff surfaces and show bias towards one lineage. CMM can be used to develop a cell culture system that could retain plasticity and target the specific lineage commitment. MSCs cultured on stiff surfaces activate YAP and the transcriptional coactivator with PDZ-binding motif (YAP/TAZ), promoting osteogenic differentiation [44]. As a result, MSCs primed on stiff surfaces increase migration even if they are rewired on a soft surface.

Cell mechanical memory manipulation has the potential of improving stem CBT, wound healing, and tissue engineering, as well as increasing our understanding about in vivo responses mediated by mechanical memory. For example, Human induced pluripotent stem cells (hiPSCs) showed reduced hepatocyte differentiation due to heterogeneity of cell populations, another consequence of maladaptation. Kim and coworkers used hyaluronic acid (HA) to block E-cadherin junctions of iPSCs [20]. The authors showed that prolonged exposure of iPSCs to HA induced cell synchronization and homogeneity. Synchronized cells were more stable and improved the capability of differentiating into hepatocytes over other possible lineages compared to untreated control cells. Therefore, synchronization driven by CMM led to a hiPSCs homogeneous population. HiPSCs’ exposure time and concentration to HA can regulate CMM and retain hiPSCs’ pluripotency. HA’s disruption of the cell-to-cell interactions caused spatial rearrangement, which benefited YAP localization. This means that when cells form a monolayer, they also have a different cytoskeletal arrangement and motility properties that could affect YAP expression compared to single cells. Many factors, including cell-to-cell interaction, cytoskeleton rearrangement, local cell density, and cell motility, play a role in the dynamic process of cellular memory.

### Fibrosis

Fibrosis is defined as damaged and scarred tissue caused by the accumulation of excess of ECM proteins and fibroblast differentiation into myofibroblasts, a process activated by the TGF- β signaling pathway. Fibrosis has been reported to underlie many age-related diseases, such as lung fibrosis [76, 77], myocardial infarctions [78], liver fibrosis [79], or CNS, and skin fibrosis [80, 81]. Lung fibrosis has a high incidence within fibrotic patients. It causes chronic skeletal muscle contraction and increases lung tissue stiffness, impairing gas exchange, and breathing difficulties.

A critical step in fibrosis research is the fibroblasts differentiation into myofibroblasts**.** All the current studies suggest that mechanical memory-based culture system could be used for stem cell expansion, preventing fibrosis in tissues, and enhancing efficacy of stem cell-based applications.

### Cellular aging

Another factor to consider is cellular aging. Aging causes senescence associated phenotypes that affect the composition of local microenvironments and activate TGF- β signaling pathway, which is responsible for regulating cell growth, differentiation, and migration. In this regard, our laboratory has shown that repeated passages on softer substrate significantly delays replicative senescence [82] and moreover, substrate stiffness also regulated the cell cycle of MSCs [83]. This suggests that mechanical memory from a soft surface may affect cellular senescence and cell cycle regulations. However, more studies are needed to test this hypothesis.

Repeated cell culture on TCP leads to maladaptation and cellular senescence due to overexpression of miRNA-21. It has been shown that erasing miRNA-21 helped to prevent fibrotic phenotype [26]. If the occurrence of replicative senescence is due to serial TCP passaging, erasing CMM should contribute to rejuvenation of senescent cells and functional improvement. Several pathologies associated with aging (i.e. fibrosis, diabetes *mellitus* Type II, arthritis) can be related to dysfunctional cellular responses promoted by chronic stress. However, these responses depend upon the cells ability to remember the stressful stimuli. Deletion of CMM in these cases can increase stress tolerance against reoccurrence and regain functional capability.

### Cancer

Cancer cells encounter diverse mechanical cues during migration and invasion. For example, breast mammary epithelial cell (MECs) progression has been correlated to the increase of tissue stiffness over time. Ondeck et al. (2019) showed that MECs cultured on a stiff/soft regulated dynamic substrate with phototunable hydrogel underwent transition from epithelial to mesenchymal phenotype through activation of YAP, TGF- β, and transcription factors like Twist1. Epithelial mesenchymal transition (EMT) and adaptability of MECs depend on the current stiffness, not on past memory [48]. Cancer cells use CMM from a primed condition, specifically, memory from a stiff environment, to metastasize in softer regions. This is a response for survival and adaptation to the opposite environment. A similar response has been observed with the oral squamous cell carcinoma (OSCC), where MCC was developed via activation of Akt and FAK pathways in response to a niche stiffness. Changes in actomyosin contractility regulated activation of CMM which promoted cancer cells to migrate and invade softer regions [19]. MCF10A cells primed on stiff surface exhibited enhanced invasion in 3D matrices [26]. Regulating CMM through alerting epigenetic processes could be a viable approach to control metastasis and develop new drugs for cancer treatment.

### Other pathologies

MCC could be manipulated in cardiomyopathies and myocardial infarctions induced by tension. Bouhrira and coworkers showed MCC in cardiac fibroblasts and cardiomyocytes cells involved in these pathological conditions [13]. MCC has been also related to regulation of osteogenic genes and activation of YAP in human periodontal ligament stem cells (hPDLSCs) [50]. For example, priming tendon cells on a physiologically soft surface could maintain the native phenotype and regulate cellular responses.

In summary, although manipulating a cell’s mechanical memory could have many beneficial implications, CMM is relatively a new concept so, it is important to continue exploring the many unknows associated to CMM outcomes before experiments can be carried out in clinical trials.

## Outlook

Recent studies on Mechanosensing and Mechanotransduction have shown that surface receptors [84], ion channels [85], actin-cytoskeletal [86], contractile machinery [87], LINC complex [88], nuclear size [89, 90], and chromatin organization [29] can display a variety of responses to mechanical stimuli. Moreover, intracellular organelles, including mitochondria, can transduce mechanical signals into genetic regulation events [91]. Therefore, understanding which functions and stimuli are mostly affected and how mechanical memory could contribute to disease and homeostasis processes would be of great interest.

The concept of CMM has permeated many research areas, including regulation of cell fate [25, 44], chromatin alteration [19, 25, 92], and cancer metastasis [19, 93]. Stem cell fate regulation could have many therapeutic implications, such as cells generation for tissue engineering, regenerative medicines, or soft tissue [3, 7, 94, 95]. This crosstalk between mechanical memory and stem cell fate regulation could be also very helpful in understanding how stem cells’ fate can be influenced by recalling past information [44]and how the switching between cellular microenvironments can contribute to the development of diseases.

**Fig. 7:**
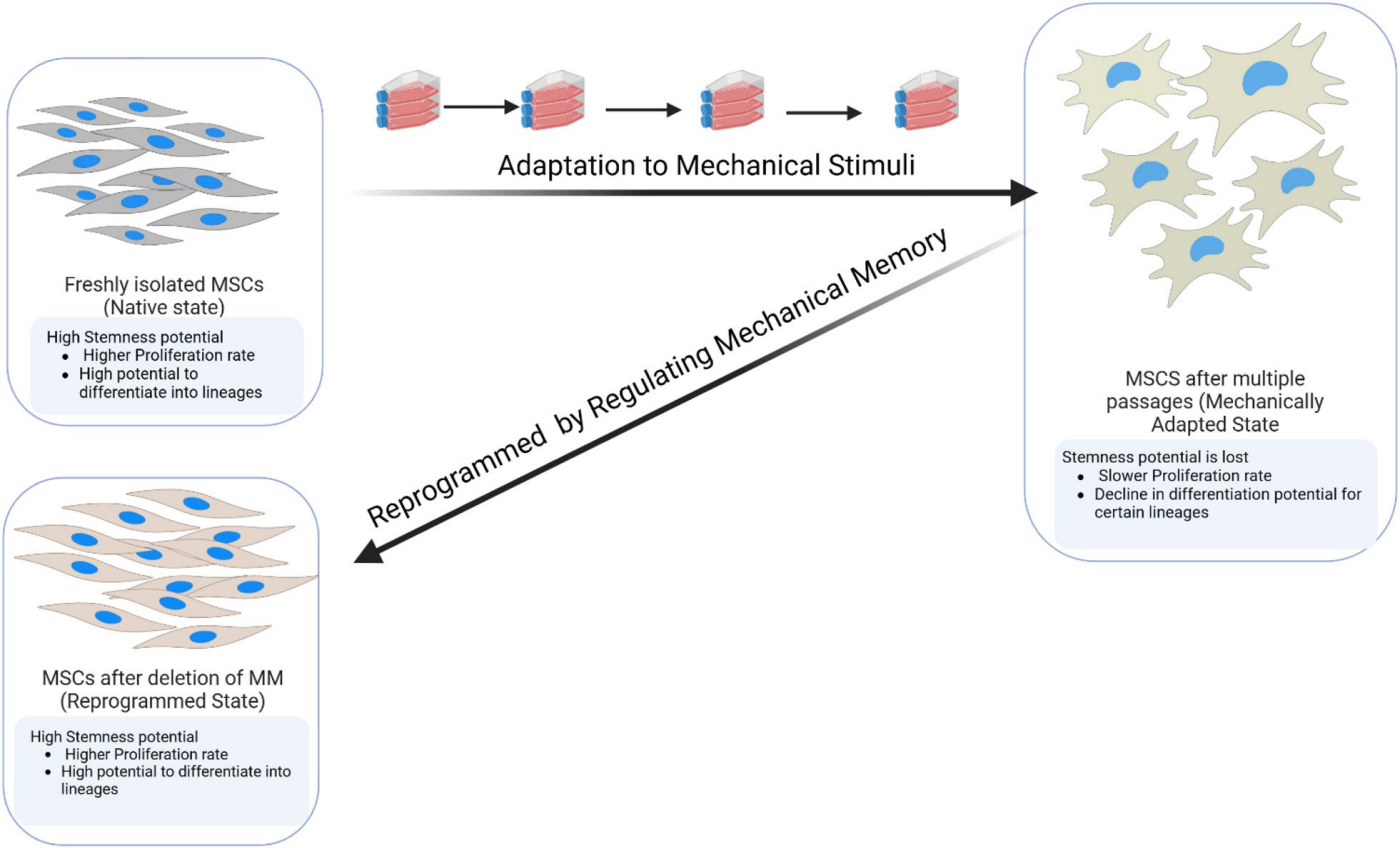
Mechanical reprogramming of MSCs. Freshly isolated MSCs or passage 0 cells are native cells and maintain their stem cell like properties. After multiple passages on cell culture plastic flasks they adapt to the the plastic stiffness. This is called  mechanically adapted state. During the adaptation they lose proliferation and differentiation potential and become not suitable for stem cell therapy. Deleting the mechanical memory acquired through an stiff surface culture either by pharmacological drugs or by mechanically exchanging the culture environment, they referred to a reprogrammed state. Therefore, MSCs can restore the lost of stemness after regulating CMM

Mechanical memory-based approaches could reverse an altered phenotype to its native cellular state with or without pharmacological drugs. In this review we have used the term cell mechano-reprogramming to define this process. For instance, manipulating the dose and duration of surface properties could reprogram the cells to their initial state (Fig. 7). Regardless of the novelty of this research area and the many breakthrough findings in the field, several questions remain to be answered (see Box 1 Outstanding Questions section). For instance, mechanical forces can influence the function of T-cells [96]. Therefore, it will be essential to understand if non-adherent cells, including immune and other stem cells, can show mechanical memory. It will also be interesting to develop new methods to better detect, understand, and be able to modify mechanical memory at the level of a single cell, which hopefully could develop into clinical applications.

Repetitive MSC culture on TCP can lead to CMM-driven maladaptation attributed to cellular senescence, reduced proliferation, and impaired differentiation. Consequently, this maladaptation can affect the regenerative ability of stem cells for preclinical and clinical applications. Senescence status is marked by impaired autophagy, increased reactive oxygen species (ROS), accumulation of dysfunctional mitochondria, and decreased telomere length [97]. Accumulation of dysfunctional mitochondria has been correlated with advancing age [98]. Investigating whether senescence and senescence markers can respond to mechanical memory modifications will be interesting.

**Fig. 8:**
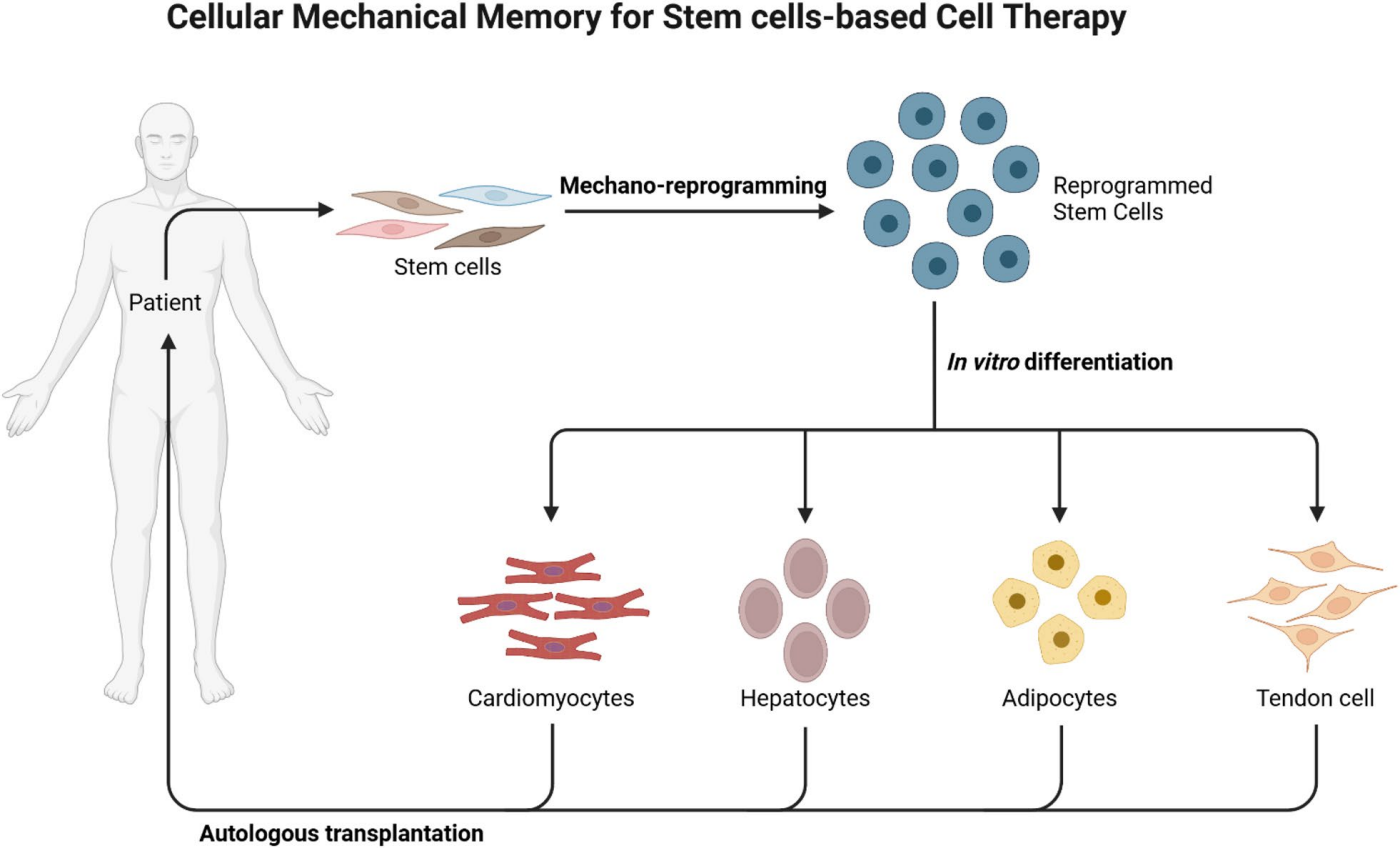
Schematic of mechano-reprogramming applications enabling control of the stem cell differentiation process within the context of stem cell therapy. The patient stem cells are collected and undergo mechano-reprogramming to erase the cell memory. This step resets the stem cells into a native state, allowing them to be differentiated into the desired lineages and ultimately be returned to the donor

Most current findings so far rely on *in vitro* results associated with CMM using substrate stiffness [8, 26, 34, 44, 99], mechanical strain [22, 100], and mechanical loading [25, 101]. However, other biophysical (mechanical) stimuli, including shear rate, optogenetics, low-frequency ultrasound, vibration, and microfluidic systems, must be investigated to continue developing strategies for new clinical applications. Only a few studies have addressed the clinical and physiological importance of the CMM [7, 34, 102]. Regulating CMM, as discussed in the previous section can be a potential strategy to mechanically reprogramming Stem cells. For instance, using dynamic cell culture systems, alternating stiff and soft surfaces could mitigate activation of CMM induced by the stiff surface. This approach could also be used to preserve the stem cell like properties and generate enough cells for therapeutic applications (Fig. 8).

Advances in the molecular identification of epigenetic factors and specific pathways involved should soon allow this goal to be achieved. CMM-based interventions could significantly improve cell-based therapies’ efficacy and outcomes in tissue regeneration and aging processes.

Outstanding questions about CMM

1. Where is the mechanical memory stored in a cell? What potential players are involved in this process?
2. Do different mechanical stimuli such as shear stress, viscoelastic surfaces, pressure pulse/ vibrations instill different mechanical memory in cells?
3. How mechanical memory of higher dose overrides the CMM on lower stimuli?
4. How swapping the mechanical environment regulates stem cell fate?
5. How does CMM affect the cell cycle?
6. Cells from soft surface lose their CMM and cells from the stiff surface retain memory, but how cells decide to retain one memory over the other?
7. In an injured environment, cells experienced a distinct condition compared to the normal healthy tissue. For example, rigidity of healthy skin is twice than the rigidity of injured tissue. Is healing of an injury regulated by the reversible nature of CMM?
8. Do suspension cells have CMM? Do they respond to swapping the microenvironment?
9. Does a cell remember a 3D microenvironment similarly to a 2D or is there any difference?

## Data Availability

References and Figures are provided.
